# Risk Stratification and Distribution of Hepatocellular Carcinomas in CEUS and CT/MRI LI-RADS: A Meta-Analysis

**DOI:** 10.3389/fonc.2022.873913

**Published:** 2022-03-29

**Authors:** Yan Zhou, Zhengyi Qin, Jianmin Ding, Lin Zhao, Ying Chen, Fengmei Wang, Xiang Jing

**Affiliations:** ^1^ School of Medicine, Nankai University, Tianjin, China; ^2^ Department of Ultrasound, Tianjin Institute of Hepatobiliary Disease, Tianjin Key Laboratory of Extracorporeal Life Support for Critical Diseases, Artificial Cell Engineering Technology Research Center, Tianjin Third Central Hospital, Tianjin, China; ^3^ Department of Gastroenterology and Hepatology, Tianjin Third Central Hospital, Tianjin, China

**Keywords:** contrast-enhanced ultrasound, contrast-enhanced magnetic resonance imaging, Contrast-enhanced computed tomography, hepatocellular carcinoma, Liver Imaging Reporting and Data System

## Abstract

**Background:**

CEUS LI-RADS and CT/MRI LI-RADS have been used in clinical practice for several years. However, there is a lack of evidence-based study to compare the proportion of hepatocellular carcinomas (HCCs) in each category and the distribution of HCCs of these two categorization systems.

**Purpose:**

The purpose of this study was to compare the proportion of HCCs between corresponding CEUS LI-RADS and CT/MRI LI-RADS categories and the distribution of HCCs and non-HCC malignancies in each category.

**Methods:**

We searched PubMed, Embase, and Cochrane Central databases from January 2014 to December 2021. The proportion of HCCs and non-HCC malignancies and the corresponding sensitivity, specificity, accuracy, diagnostic odds ratio (DOR), and area under the curve (AUC) of the LR-5 and LR-M categories were determined using a random-effect model.

**Results:**

A total of 43 studies were included. The proportion of HCCs in CEUS LR-5 was 96%, and that in CECT/MRI LR-5 was 95% (*p* > 0.05). The proportion of non-HCC malignancy in CEUS LR-M was lower than that of CT/MRI LR-M (35% vs. 58%, *p* = 0.01). The sensitivity, specificity, and accuracy of CEUS LR-5 for HCCs were 73%, 92%, and 78%, respectively, and of CT/MRI LR-5 for HCCs, 69%, 92%, and 76%, respectively.

**Conclusion:**

With the upshift of the LI-RADS category, the proportion of HCCs increased. CEUS LR-3 has a lower risk of HCCs than CT/MRI LR-3. CEUS LR-5 and CT/MRI LR-5 have a similar diagnostic performance for HCCs. CEUS LR-M has a higher proportion of HCCs and a lower proportion of non-HCC malignancies compared with CT/MRI LR-M.

## Highlights

▪ CEUS LR-3 has a lower proportion of HCCs than CT/MRI LR-M, while CEUS LR-M has a higher proportion of HCCs.
▪ Most of HCCs are in CEUS LR-5, LR-M, and LR-4, while most of HCCs are in CT/MRI LR-5 and LR-4.
▪ CEUS LR-M has a lower proportion of non-HCC malignancies but a higher proportion of HCCs compared with CT/MRI LR-M.

## Introduction

Contrast-enhanced computed tomography (CT), contrast-enhanced magnetic resonance imaging (MRI), and contrast-enhanced ultrasound (CEUS) were recommended by international guidelines to diagnose hepatocellular carcinomas (HCCs) (1, 2). To standardize the terminology, techniques, interpretation, reporting, and data collection of liver imaging, the American College of Radiology (ACR) released CT/MRI and the CEUS Liver Imaging Reporting and Data System (LI-RADS) (3, 4).

The contents of LI-RADS include the application of LI-RADS, techniques in different contrast-enhanced examinations, categorization, and management of lesions. According to the lesion size, major features, and ancillary features, lesions can be classified into different categories, including LR-1 to LR-5, LR-M, LR-TIV, and LR-NC. The clinical management for lesions of these categories was suggested by ACR LI-RADS based on the empirical risk of HCCs or malignancies. For example, lesions categorized into LR-5 can go through the management of HCCs without biopsy.

CT/MRI and CEUS LI-RADS were two independent systems with the same strata of categorization. Among LR-1 to LR-5 and LR-M, lesions in CEUS LI-RADS and CT/MRI LI-RADS have similar suggested management except for LR-3. This is because the positive predictive value (PPV) of HCCs is higher in CEUS LR-3 than that in CT/MRI LR-3 (3–6). Thus, the multidisciplinary discussion (MDD) was suggested additionally in CEUS LR-3. Otherwise, CT/MRI and CEUS LI-RADS are hypothesized to correspond to the same risk of HCCs in other categories. Up to now, there is a lack of evidence-based study to compare the proportion of HCCs in other categories and the distribution of HCCs of these two categorization systems (7–9). Moreover, whether the categories corresponding to similar suggestions of managements in CT/MRI LI-RADS and CEUS LI-RADS have a similar risk of HCCs is still unclear, which implies whether the suggested management is appropriate for LI-RADS categories also remains to be verified. In this meta-analysis, therefore, we aim to explore the risk and distribution of HCCs and non-HCC malignancies in each category of CEUS and CT/MRI LI-RADS and to explore the diagnostic performance of HCCs by LR-5 and of non-HCC malignancies by LR-M.

## Method

This meta-analysis was conducted following the Preferred Reporting Items for Systematic Reviews and Meta-analysis (PRISMA) Statement (10). This study was registered at the Prospero International Prospective Register of Systematic Reviews (CRD42020175800).

### Literature Search Strategy

We searched corresponding studies from January 2014 to December 2021 in the PubMed, Embase, and Cochrane Central databases. The details of the strategy of searching are provided in **Supplementary Table 1**. Only English articles were included in this study. Case reports, reviews, letters, comments, and erratum were excluded.

### Inclusion and Exclusion Criteria

We included studies that met the following criteria: (1) patients with high risk for HCC; (2) the observations undergoing contrast-enhanced CT/MRI examination categorized according to CT/MRI LI-RADS V2014, V2017, or V2018, or the observations undergoing CEUS classified according to CEUS LI-RADS V2016 or V2017; (3) the contrast agent for CEUS being SonoVue; and (4) pathology or composite clinical reference standard (CCRS, multiple imaging or imaging follow-up) used as the reference standard. The exclusion criteria were as follows: (1) studies applied to patients without high risk for HCCs, (2) studies including duplicated data, (3) studies only including HCCs or HCCs and non-HCC malignancies, and (4) studies without sufficient data for inclusion in the pooled analysis.

### Study Selection

After excluding duplicates, two researchers independently reviewed the titles and abstracts of the articles. The full texts of the relevant articles were read to determine their inclusion. In the case of multiple studies from a center, we selected the most recent and complete one.

### Data Extraction

The following data were extracted from the included studies: (1) the characteristics of the study, including the first author, year of publication, nationality of patients, time of patient recruitment, and design (prospective or retrospective); (2) the characteristics of patients, including the number of patients, ages, and sexes; and (3) the tests to be evaluated, reference criteria, and results. The number of observations, HCCs, non-HCC malignancies, and benign lesions in each LI-RADS category was extracted from each study. If more than one data set was available in a study (e.g., different data from more than one viewer), the average data were adopted. Data extraction was conducted independently by the aforementioned two researchers, and no discrepancy was found during the process.

### Quality Assessment

The Quality Assessment of Diagnostic Accuracy Studies (QUADAS-2) tool (11) was used to evaluate the research Quality. QUADAS-2 includes four aspects: patient selection, index test, reference standard, flow, and timing. For each aspect, the risk of bias was classified as high, low, or unknown. Two researchers independently assessed the risk of bias for each study, and any discrepancy was resolved by discussion with the third researcher. The results of the risk of bias assessments are shown in **Supplementary Figure 1**.

### Statistical Analysis

Random-effect models were used to evaluate the proportion of HCCs and non-HCC malignancies in each LI-RADS category, and the sensitivity, specificity, accuracy, and diagnostic odds ratio (DOR) of the LR-5 and LR-M categories, and to generate forest plots and 95% confidence intervals (95% CIs). The Q test and I^2^ statistic were used to analyze the heterogeneity of the study, and I^2^ >50% was considered to indicate heterogeneity (12). The variance of the logit-transformed percentage method was used to compare the differences in the proportions of HCCs and non-HCC malignancies in each category, and in the sensitivity, specificity, accuracy, and DOR of LR-5/M. The publication bias of the proportion of HCCs and non-HCC malignancies in each category was not evaluated according to the guidance of diagnostic test accuracy of systematic reviews (10). All statistical analyses were performed by the R language (v3.6.3, R Foundation for Statistical Computing, Vienna, Austria).

## Results

**Figure 1:**
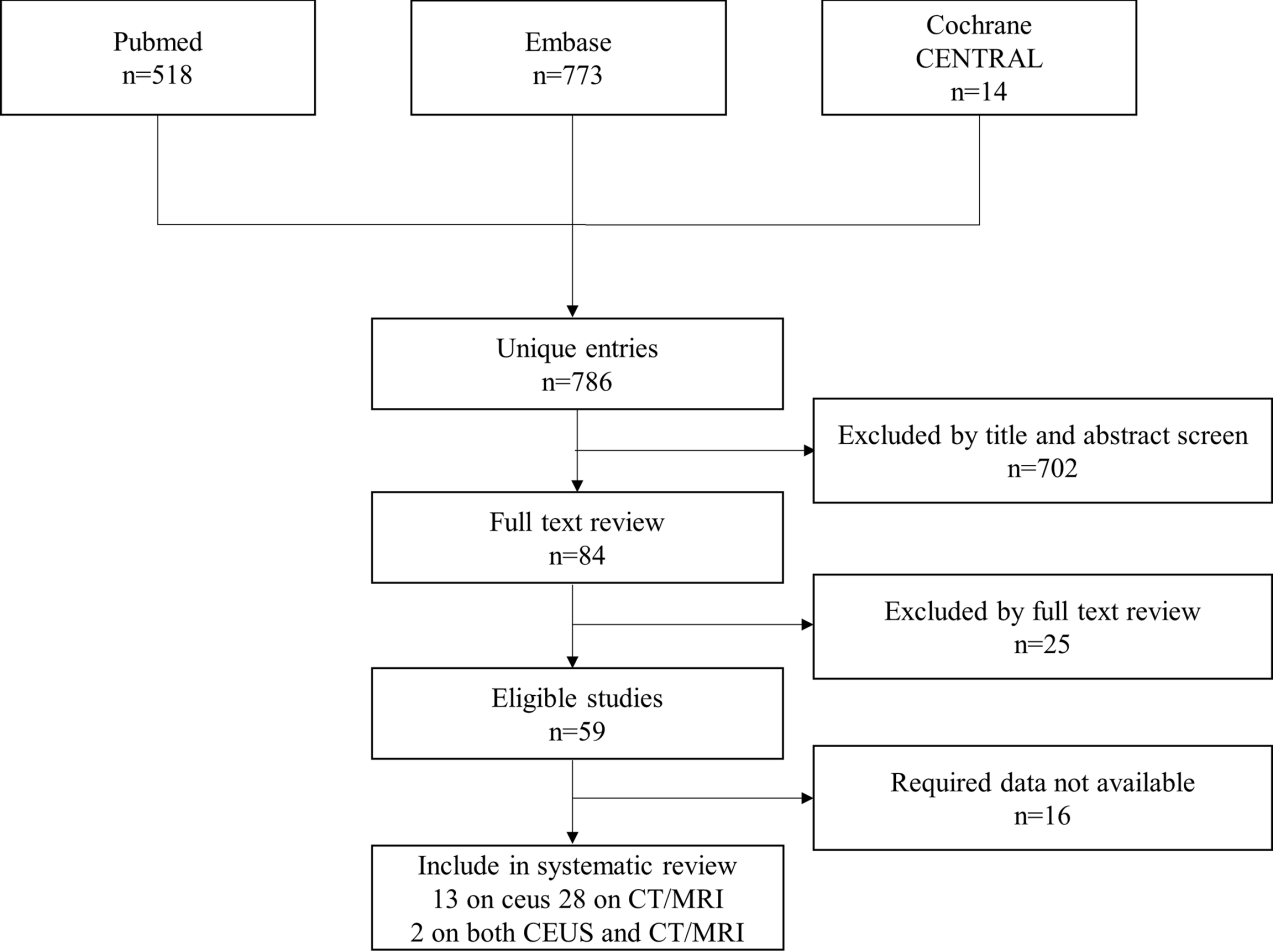
Flow diagram of the selection of studies.

A total of 786 studies were initially identified. 84 studies were then reviewed, and 59 studies were considered suitable for inclusion in this meta-analysis. After further excluding studies with insufficient data in the analysis, 43 studies were finally included (**Figure 1**) (13–55). Detailed information of the included and excluded studies is shown in **Supplementary Tables 2, 3**.

There were 15 studies on CEUS LI-RADS involving 6,573 patients with 7,234 lesions, including 5,387 HCCs, 624 non-HCC malignancies, and 1,223 benign lesions. There were 30 studies on CT/MRI LI-RADS involving 5,274 patients with 6,522 lesions, including 4,554 HCCs, 481 non-HCC malignancies, and 1,487 benign lesions.

### Proportions and Distribution of HCCs in Each CEUS and CT/MRI LI-RADS Category

**Figure 2:**
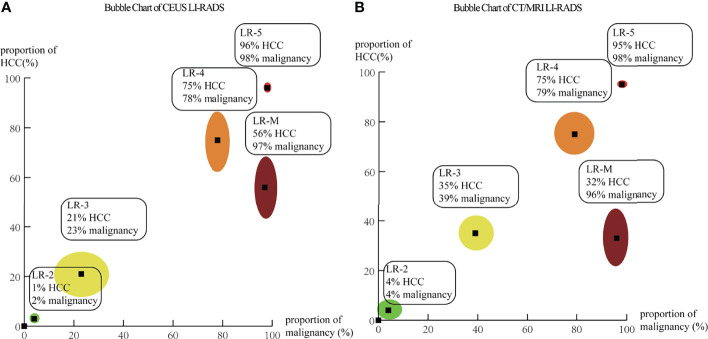
Bubble chart based on pooled percentage of HCCs and non-HCC malignancies for each category of CEUS **(A)** and CT/MRI **(B)** LI-RADS. The points at the centers of bubbles correspond to the pooled percentages of HCCs and non-HCC malignancies. The outer bubble margins correspond to 95% CIs for percentages of HCCs (y-axis) and non-HCC malignancies (x-axis).

A total of 5,387 HCCs in CEUS and 4,554 HCCs in CT/MRI can be used for the calculation of proportions and distribution of HCCs in each CEUS and CT/MRI LI-RADS category. There is no HCC in the CEUS and CT/MRI LR-1. The proportion of HCC gradually increases with the upshift of the category of both CEUS and CT/MRI LI-RADS. The proportions of HCCs of LR-2, 3, 4, and 5 were 1%, 21%, 75%, and 96% for CEUS LI-RADS and 4%, 35%, 75%, and 95% for CT/MRI LI-RADS, respectively. The proportion of HCCs in CEUS LR-3 is lower than that of CT/MRI LR-3 (21% vs. 35%, *p* = 0.02). The proportion of HCCs in CEUS LR-M is 56% (95% CI: 44%–69%), which is higher than that in CT/MRI LR-M, namely, 33% (95% CI: 22%–45%) (*p* = 0.01). The proportion of HCCs in each category is shown in **Table 1** and **Figure 2**. The forest plots of percentages of HCCs in CEUS and CT/MRI LR-5 are provided **Supplementary Figure 2**.

**Table 1:** Proportions of HCCs in each CEUS and CT/MRI LI-RADS category.

	CEUS			CT/MRI			
	HCC (95% CI)	Observations	I^2^, %	HCC (95% CI)	Observations	I^2^, %	p
LR-2	1 (0–6)	134	0	4 (1–9)	297	57	0.33
LR-3	21 (13–31)	670	78	35 (29–43)	835	73	0.02
LR-4	75 (61–85)	735	88	75 (65–82)	1299	89	0.99
LR-5	96 (94–98)	3858	89	95 (93–97)	3205	83	0.46
LR-M	56 (44–69)	1361	93	33 (22–45)	490	80	0.01
LR-TIV	97 (77–100)	100	0	72 (58–83)	103	13	0.03

**Figure 3:**
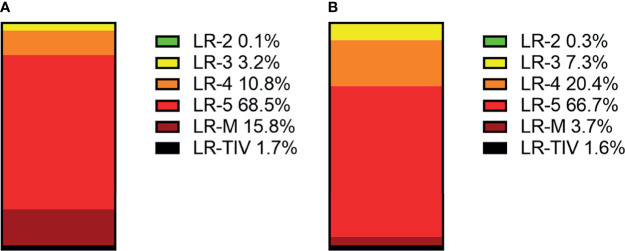
Distributions of HCCs in each CEUS category **(A)** and CT/MRI category **(B)**.

In CEUS LI-RADS, most of the HCCs are in LR-5 (68.5%). Most of the rest HCCs are in LR-M (15.8%) and LR-4 (10.8%). In CT/MRI LI-RADS, most of the HCCs are also in LR-5 (66.7%). Most of the remaining HCCs are in LR-4 (20.4%) but not LR-M (3.7%). There are more HCCs classified into CT/MRI LR-2, 3, and 4, compared with CEUS LR-2, 3, and 4, while there are more HCCs classified into CEUS LR-M than CT/MRI LR-M. The distributions of HCCs among different categories are shown in **Figure 3** and **Supplemental Table 4**.

### Proportions and Distribution of Non-HCC Malignancies in Each CEUS and CT/MRI LI-RADS Category

A total of 624 non-HCC malignancies in CEUS and 481 non-HCC malignancies in CT/MRI can be used for the calculation of proportions and distributions of non-HCC malignancies in each CEUS and CT/MRI LI-RADS category. There is no non-HCC malignancy in the CEUS and CT/MRI LR-1. The proportions of non-HCC malignancies in CEUS and CT/MRI LR-2 to LR-5 range from 1% to 5%. The proportion of non-HCC malignancies in CEUS LR-M is 35%, significantly lower than that of CT/MRI LR-M (58%, *p* = 0.01). The proportions of non-HCC malignancies among different categories are shown in **Table 2**. The forest plots of percentages of non-HCC malignancies in CEUS and CT/MRI are depicted in **Supplementary Figure 3**.

**Table 2:** Proportions of non-HCC malignancies in each CEUS and CT/MRI LI-RADS category.

	CEUS			CT/MRI			
	Malignancy (95% CI)	Observations	I^2^, %	Malignancy (95% CI)	Observations	I^2^, %	p
LR–2	4 (1-11)	126	0	5 (3–9)	294	0	0.73
LR–3	5 (2–11)	644	52	4 (3–6)	766	0	0.70
LR–4	1 (0–6)	629	80	3 (1–4)	1266	58	0.40
LR–5	2 (1–3)	3594	82	2 (1–3)	2993	68	0.65
LR–M	35 (26–45)	1323	90	58 (43–72)	441	85	0.01
LR–TIV	3 (0-23)	100	62	22 (11–40)	102	43	0.08

**Figure 4:**
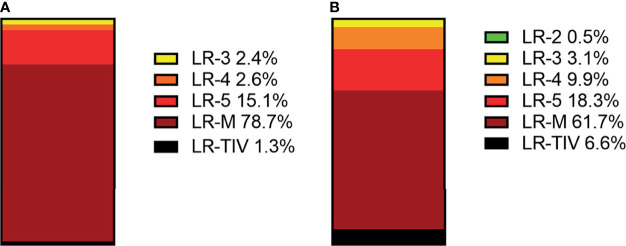
Distributions of non-HCC malignancies in each CEUS category **(A)** and CT/MRI category **(B)**.

In CEUS LI-RADS, most of the non-HCC malignancies are in LR-M (78.7%), most of the remaining non-HCC malignancies are in LR-5 (15.1%). In CT/MRI LR-RADS, most of the non-HCC malignancies are in LR-M (61.7%), and most of the remaining non-HCC malignancies are in LR-5 (18.3%) and LR-4 (9.9%). The distributions of non-HCC malignancies among different categories are shown in **Figure 4** and **Supplementary Table 5**.

### Meta-Regression for the Proportion of HCCs in Each LI-RADS Category

We analyzed the population (Asian or non-Asian), the reference standard (pathological or CCRS), and the version of LI-RADS used in these studies. The meta-regression results show that the proportion of HCCs in LR-3 for Asians is lower than that of LR-3 for non-Asians (14.3% vs. 32.3%, *p* = 0.02). The proportion of HCCs in CEUS LR-M for Asian is higher than that of LR-M for non-Asian (67.3% vs. 35.7%, *p* < 0.01).

The meta-regression results for CT/MRI LI-RADS show that the proportions of HCCs in CT/MRI LR-4 and CT/MRI LR-5 using pathology as the reference standard are lower than those using CCRS as the reference standard (for LR-4: 63.4% vs. 81.2%, *p* = 0.03; for LR-5: 92.3% vs. 97%, *p* = 0.01). The proportions of HCCs in CT/MRI LR-M using LI-RADS V2017 are lower than that using V2018 (6% vs. 42.2%, *p* = 0.04).

### Diagnostic Performance of LR-5 for HCCs

**Figure 5:**
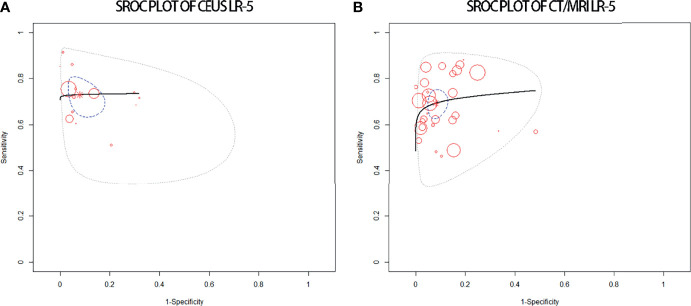
sROC plots of CEUS LR-5 **(A)** and of CT/MRI LR-5 **(B)** for the diagnosis of HCCs.

The pooled sensitivity, specificity, and accuracy of CEUS LR-5 for HCC are comparable to those of CT/MRI LR-5 (**Table 3**). The DOR and the area under the summary receiver operating characteristic (SROC) curve for CEUS LR-5 are 28.0 and 0.74, and for CT/MRI LR-5, 23.9 and 0.75, as depicted in **Figure 5**.

**Table 3:** Diagnostic performance of CEUS and CT/MRI LR-5 for HCCs.

		CEUS	I^2^,%	CT/MRI	I^2^,%	p
LR-5	Sensitivity(95% CI)	73% (67–78)	87	69% (64–74)	92	0.32
	Specificity(95% CI)	92% (86–95)	75	92% (88–94)	86	0.96
	Accuracy(95% CI)	78% (71–84)	90	76% (72–79)	93	0.54
	DOR (95% CI)	28.0 (14.2–55.3)	79	23.9 (15.8–36.3)	87.3	0.70
	AUC	0.74		0.75		

### Diagnostic Performance of LR-M for Non-HCC Malignancies

**Figure 6:**
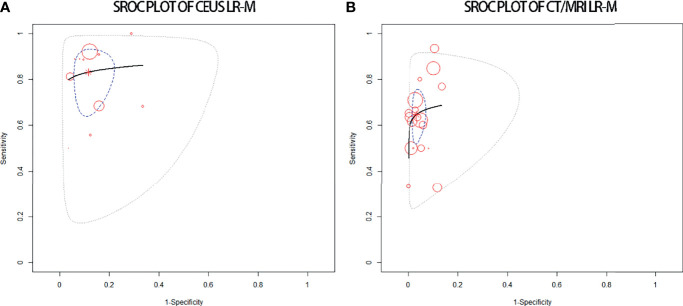
sROC plots of CEUS LR-M **(A)** and of CT/MRI LR-M **(B)** for the diagnosis of non-HCC malignancies.

The pooled sensitivity for non-HCC malignancies in CEUS LR-M (83%) is higher than that of CT/MRI LR-M (65%), while the pooled specificity for non-HCC malignancies in CT/MRI LR-M (92%) is similar to that of CEUS LR-M (92%) (**Table 4**). The DOR and the area under the SROC curve for CEUS LR-M are 36.5 and 0.87, for CT/MRI LR-M 46.6 and 0.73, respectively, as depicted in **Figure 6**.

**Table 4:** Diagnostic performance of CEUS and CT/MRI LR-M for non-HCC malignancies.

		CEUS	I^2^,%	CT/MRI	I^2^,%	p
LR-M	Sensitivity (95% CI)	83% (73–90)	53	65% (56–73)	78	0.01
	Specificity (95% CI)	92% (86–95)	75	92% (88–94)	86	0.96
	Accuracy (95% CI)	78% (70–84)	90	76% (72–79)	93	0.54
	DOR (95% CI)	36.5 (16.6–80.0)	96	46.6 (24.9–88.2)	86	0.64
	AUC	0.87		0.73		

### Meta-Regression for the Diagnostic Performance

The meta-regression for CT/MRI LR-5 shows that the specificity of the studies using pathology as the reference standard is lower than that using CCRS (85.4% vs. 95.0%, *p* < 0.01).

CEUS LR-M V2017 has a higher sensitivity (85.1% vs. 78.0%, *p* < 0.01), lower specificity (85.8% vs. 96.0%, *p* < 0.01), and lower accuracy (84.9% vs. 95.0%, *p* < 0.01) for the diagnosis of non-HCC malignancies compared with that of CEUS LR-M V2016. The sensitivity, specificity, and accuracy of CEUS LR-M for the diagnosis of non-HCC malignancies in the studies using pathology as the reference standard are lower compared with those using CCRS as the reference (sensitivity: 60.0% vs. 69.0%, *p* = 0.01; specificity: 96.0% vs. 97.2%, *p* = 0.02; accuracy: 90.1% vs. 94.63%, *p* < 0.01). Meta-regression for specificity shows that CT/MRI LR-M V2017 has a higher specificity than CT/MRI LR-M V2018 (99.5% vs. 93.4%, *p* < 0.01) for the diagnosis of non-HCC malignancies. CT/MRI LR-M V 2017 has a higher accuracy than CT/MRI LR-M V2014 (96.9% vs. 93.2%, *p* = 0.02) and V2018 (96.9% vs. 90.1%, *p* < 0.01).

## Discussion

To our best knowledge, this work represents the first systematic review of the comparison of the percentages and distributions of HCCs and non-HCC malignancies between the CEUS and CT/MRI LI-RADS. The upshift of LI-RADS categories from LR-1 to LR-5 mirrors monotonically greater proportions of HCCs. We found that the proportion of HCCs in CEUS LR-3 is lower than that of CT/MRI LR-3. However, the proportions of HCCs in CEUS LR-M are higher than those of CT/MRI LI-RADS, while the percentage of non-HCC malignancies in CEUS LR-M is lower than that of CT/MRI LR-M. Furthermore, CEUS LR-M has a higher sensitivity in the diagnosis of non-HCC malignancies than CT/MRI LR-M.

ACR LI-RADS aims at stratifying the risk of HCCs and recommending the clinical management of each category (3, 4). The risk of HCCs in each category is the basis of clinical management. Evidence-based studies and feedback from clinical practice can help advise on the classification and management of lesions. ACR published two LI-RADS systems, namely, CEUS LI-RADS and CT/MRI LI-RADS. Although CEUS LI-RADS and CT/MRI LI-RADS have the same categories, the two LI-RADS systems have differences among the criteria and managements of classifications (3, 4). Understanding whether corresponding categories of the two LI-RADS systems lead to distinct risk stratification of HCCs and whether the recommended management is appropriate for each category is of paramount importance. However, there is no evidence-based systematic review to address the issues mentioned above.

In this systematic review, we found that there was no statistical significance in the proportions of HCCs of the corresponding CEUS and CT/MRI LI-RADS categories, except CEUS LR-3 and LR-M and the CT/MRI counterparts. On the one hand, there is no HCC or non-HCC malignancy in the CEUS and CT/MRI LR-1, which is consistent with the definition of LR-1, i.e., definite benign. On the other hand, there are 96% HCCs in CEUS LR-5 and 95% HCCs in CT/MRI LR-5, which is also consistent with the definition of LR-5, definite HCCs. Thus, the lesions in CEUS LR-5 or CT/MRI LR-5 can go through clinical management of HCC without biopsy or MDD, as suggested by ACR. The management of CEUS LR-3, however, was different from that of CT/MRI LR-3. The suggested management for CT/MRI LR-3 is alternative or repeating diagnostic imaging in 3–6 months. By comparison, the suggested management for CEUS LR-3 is alternative or repeating diagnostic imaging in ≤6 months, with consideration for MDD. The recommended management for CEUS LR-3 is based on retrospective studies, which demonstrate that the percentage of CEUS LR-3 is 60%, higher than that of CT/MRI LR-3 (56–58). In the present study, the pooled proportion of HCCs in CEUS LR-3 is lower than that of CT/MRI LR-3 (21% vs. 35%, *p* = 0.02), which implies that there is still space for future improvement of the suggested management for CEUS and CT/MRI LR-3.

One of the main goals of LI-RADS LR-M is to avoid misdiagnosis of hepatocellular carcinoma for cholangiocarcinoma. In this study, we found that the percentage of HCCs in CEUS LR-M is higher than that of CT/MRI LR-M (56% vs. 33%). This difference may be induced by the differences in the diagnostic criteria of LR-M between CEUS and CT/MRI LI-RADS. Lesions with rim APHE or early washout or marked washout are classified into CEUS LR-M. Part of HCCs, especially the moderately and poorly differentiated HCCs, can present imaging features of LR-M (59). Compared with the suggested management of LR-5, MDD, alternative or repeating imaging, biopsy, or treatment is needed additionally for LR-M. Currently, lesions in CEUS LR-M have the same recommended management as those in CT/MRI LR-M. Thus, part of HCCs in CEUS LR-M, which can go through treatment, still needs an additional examination or MDD in practice. Despite the higher proportion of HCCs and the lower proportion of non-HCC malignancies in CEUS LR-M, the PPV and specificity of HCCs in CEUS LR-5 and CT/MRI LR-5 were comparable, which means that CEUS LR-M can avoid misdiagnosis of HCCs for cholangiocarcinoma. Still, in order to reduce the proportions of HCCs with additional examination or MDD, a previous study aimed at withdrawing HCCs in CEUS LR-M to LR-5 without decreasing the positive predictive value and specificity of HCCs in CEUS LR-5 (17).

LI-RADS LR-5 is used as the diagnostic criteria for HCCs, and LR-M is used as the diagnostic criteria for non-HCC malignancies in some studies (59, 60). The results of our systematic review show that CEUS LR-5 and CT/MRI LR-5 have comparable diagnostic performance for HCCs, namely, similar sensitivity, specificity, and accuracy. Our results are consistent with the result of previous studies (61). For non-HCC malignancies, however, CEUS LR-M has a different risk and sensitivity compared with CT/MRI LR-M. At the beginning of the application of CEUS LI-RADS, some studies focused on the PPV of LR-M in the diagnosis of non-HCC malignancies and found that CEUS LR-M has lower PPV than CT/MRI LR-M. They concluded that CT/MRI LR-M has higher differential diagnostic performance for non-HCC malignancies compared with CEUS LR-M (20, 53). Hu et al. (62) compared the diagnostic performance of non-HCC malignancies between CEUS LR-M and CT/MRI LR-M and demonstrated that the two LI-RADS systems had similar performance and sensitivity. However, a meta-analysis from the same group found that CEUS LR-M has a high sensitivity (84%) and specificity (90%) for non-HCC malignancies, while the CT/MRI counterpart has a moderate sensitivity (63%) and high specificity (95%) (61). In this study, CT/MRI LR-M has a higher percentage of non-HCC malignancies compared with CEUS LR-M, in agreement with previous studies (9, 63). CEUS LR-M, however, has higher sensitivity of non-HCC malignancies compared with CT/MRI LR-M. Thus, we conclude that both of the two LI-RADS systems have their advantages for the differential diagnosis of HCCs and non-HCC malignancies. Further studies are needed to explore the diagnostic performance for non-HCC malignancies.

This study has several limitations. First, we aimed to compare the risk of HCCs for CEUS LI-RADS classifications and the CT/MRI counterparts. However, few paired studies are available for this review. Second, the heterogeneity of the distribution and diagnostic performance of HCCs cannot be well explained by the meta-regression analysis. Last, the effects of tumor size on the classification by LI-RADS were not explored.

In conclusion, the proportions of HCCs increase with the upshift of LI-RADS categories from LR-1 to LR-5. CEUS LR-3 has a lower proportion of HCCs than CT/MRI LR-3, while CEUS LR-M has a higher proportion of HCCs. CEUS LR-M has a lower proportion of non-HCC malignancies than CT/MRI LR-M. CEUS LR-5 and CT/MRI LR-5 show comparable diagnostic performances of HCC, while CEUS LR-M has a higher sensitivity of non-HCC malignancies compared with CT/MRI LR-M.

## Data Availability Statement

The original contributions presented in the study are included in the article/**Supplementary Material**. Further inquiries can be directed to the corresponding authors.

## Author Contributions

YZ and ZQ designed the study and wrote the manuscript. JD, YC, and LZ collected the data. XJ and FW supervised the findings of this study. All authors contributed to the article and approved the submitted version.

## Funding

The present work was supported by the Tianjin Health and Health Committee (Nos. MS20017, KJ20170, ZD20014,NQ20033) and founded by the Tianjin Key Medical Discipline (Specialty) Construction Project.

## Conflict of Interest

The authors declare that the research was conducted in the absence of any commercial or financial relationships that could be construed as a potential conflict of interest.

## Publisher’s Note

All claims expressed in this article are solely those of the authors and do not necessarily represent those of their affiliated organizations, or those of the publisher, the editors and the reviewers. Any product that may be evaluated in this article, or claim that may be made by its manufacturer, is not guaranteed or endorsed by the publisher.

## Abbreviations

HCC, hepatocellular carcinoma; LI-RADS, Liver Imaging Reporting and Data System; CEUS, contrast-enhanced ultrasonography; MRI, magnetic resonance imaging; CT, computed tomography; ACR, American College of Radiology; PPV, positive predictive value; CCRS, composite clinical reference standard.
