# Supplementary material for: Risk Stratification and Distribution of Hepatocellular Carcinomas in CEUS and CT/MRI LI-RADS: A Meta-Analysis
Source: Front Oncol. 2022 Mar 29;12:873913. doi: 10.3389/fonc.2022.873913 (PMC9001845; doi:10.3389/fonc.2022.873913)
Supplement: Supplementary file 1 [file DataSheet_1.docx]

Supplementary Information for

Risk stratification and distribution of hepatocellular carcinomas in CEUS and CECT/MRI LI-RADS: A meta-analysis

Yan Zhou^†^ MD; Zhengyi Qin^†^, MSc; Jianmin Ding, MD; Lin Zhao, PhD; Ying Chen, MD; Fengmei Wang, PhD, Xiang Jing, MD

From School of Medicine, Nankai University, Tianjin 300071, China(Y.Z., F.W.); Department of Ultrasound, Tianjin Institute of Hepatobiliary Disease, Tianjin Key Laboratory of Extracorporeal Life Support for Critical Diseases, Artificial Cell Engineering Technology Research Center, Tianjin Third Central Hospital, Tianjin, China (Y.Z., Z.Y., J.D., L.Z., Y.C., X.J.); Department of Gastroenterology and Hepatology, Tianjin Third Central Hospital, Tianjin, China (F.W.)

Tianjin Third Central Hospital, Hedong District, No. 83 Jintang Road, Tianjin, China

†These authors have contributed equally to this work and share first authorship

Corresponding author: Xiang Jing, Fengmei wang

Tel:+86 022 84112323;

E-mail:dr.jingxiang@aliyun.com;

Address: Hedong District, No. 83 Jintang Road, Tianjin, China, 300170

**Supplementary Figures**


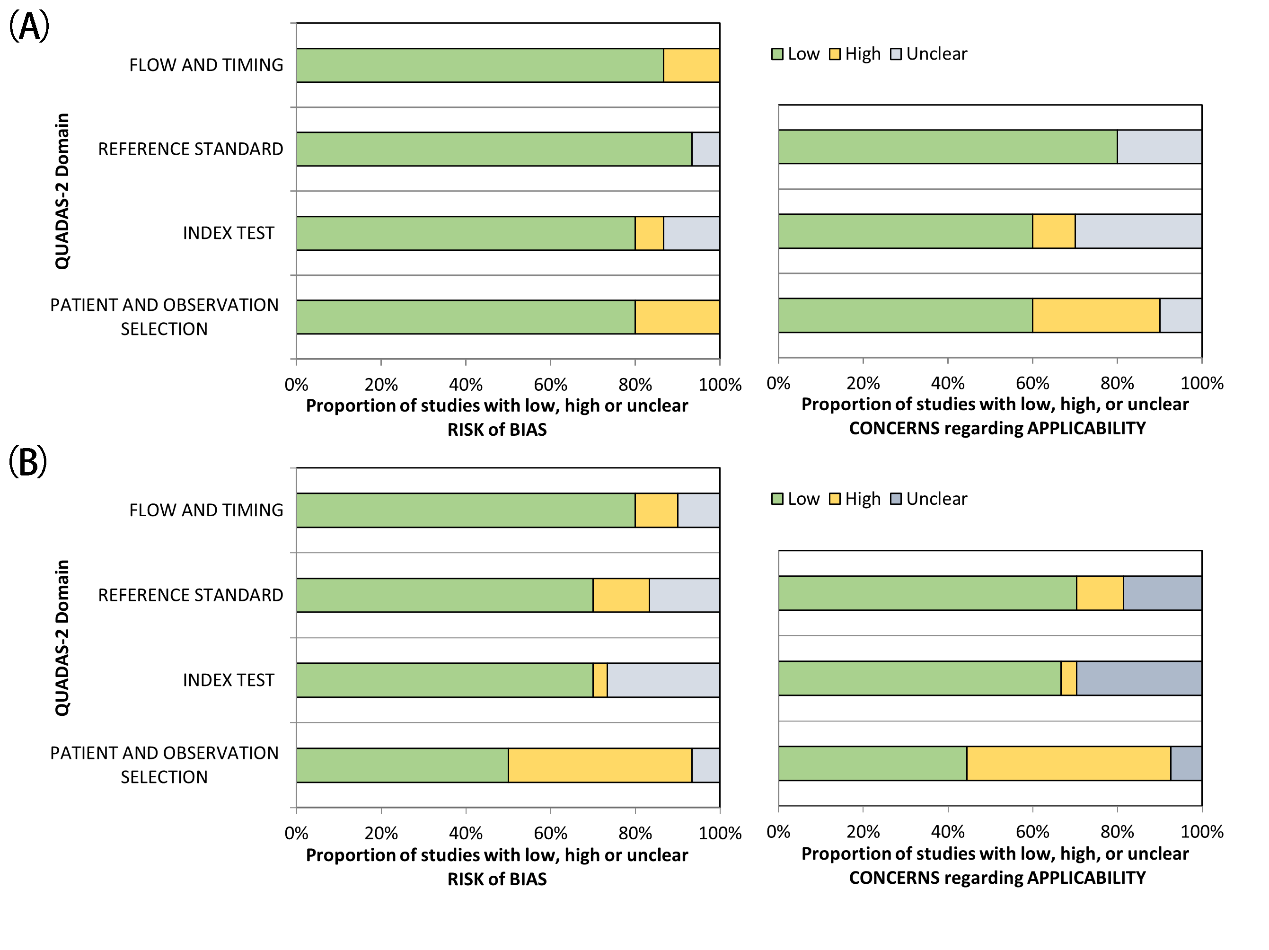


Figure 1: Methodological quality of the studies with the Quality Assessment of Diagnostic Accuracy Studies (QUADAS)-2. (A)Studies on CEUS-LI-RADS. (B)Studies on CT/MRI-LI-RADS. The results of the included studies in terms of the risk of bias (left) and concerns regarding applicability (right) according to each QUADAS-2 domain.


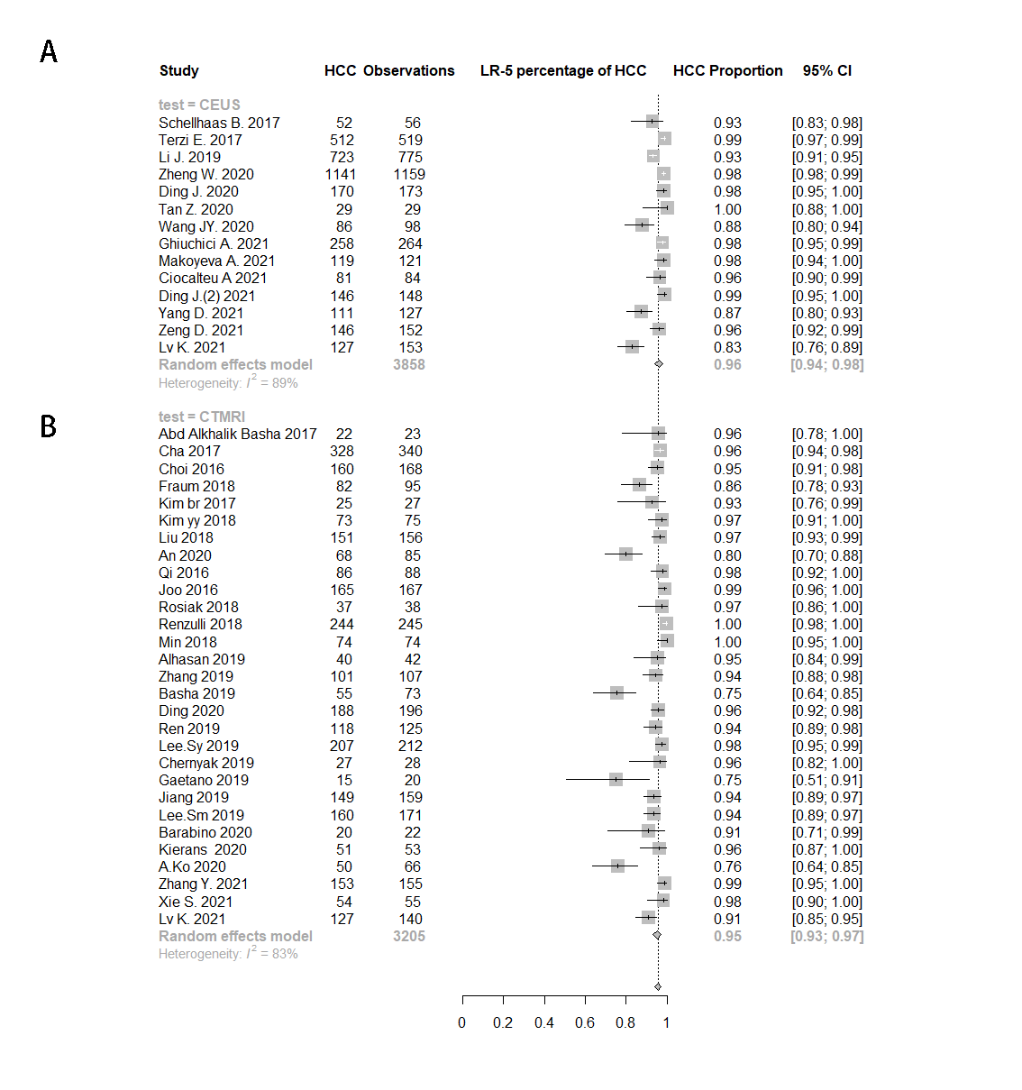


Figure 2: (A) The forest plots of percentage of HCC in CEUS LR-5. (B) The forest plots of percentage of HCC in CT/MRI LR-5.


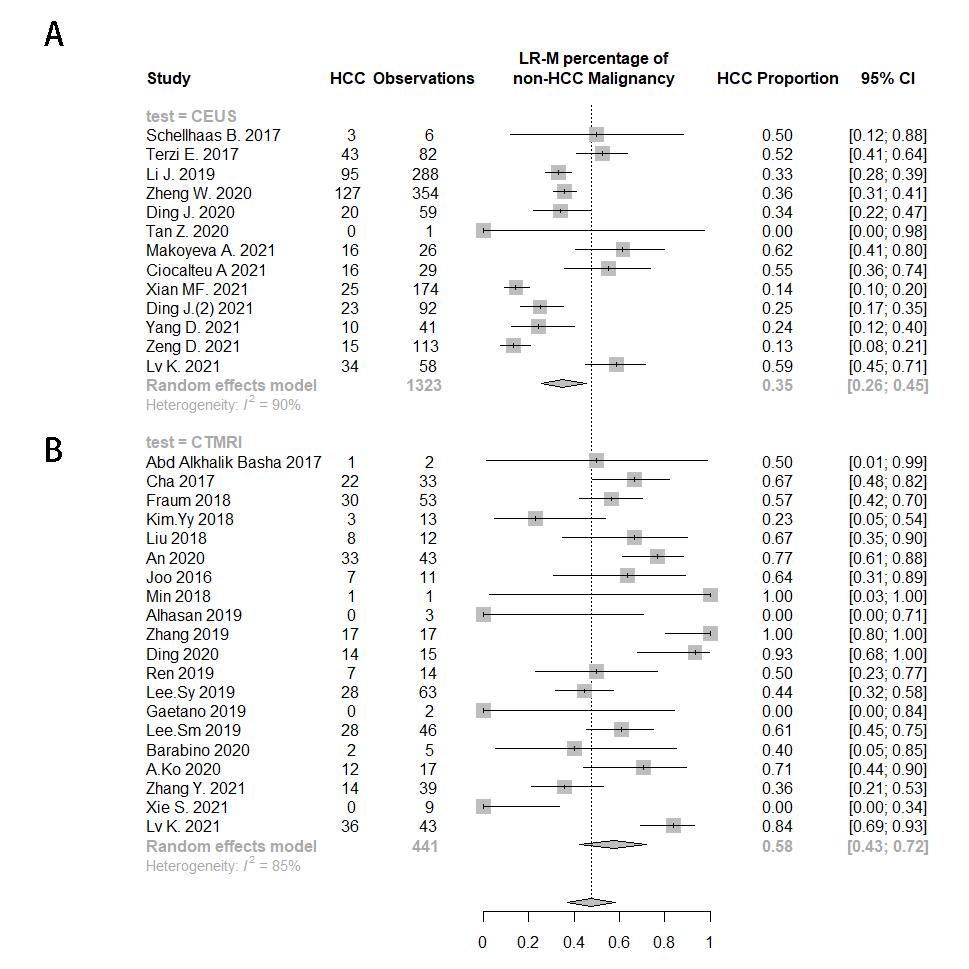


Figure 3 (A) The forest plots of percentage of non-HCC malignancies in CEUS LR-M. (B) The forest plots of percentage of non-HCC malignancies in CT/MRI LR-M.

Table 1: Literature search strategy

| Database | search strategy |
| --- | --- |
| Pubmed | ((((((((liver imaging reporting and data system[Title/Abstract]))) OR ((LI-RADS[Title/Abstract] OR LIRADS[Title/Abstract]))) OR ((LR-1[Title/Abstract] OR LR-2[Title/Abstract] OR LR-3[Title/Abstract] OR LR-4[Title/Abstract] OR LR-5[Title/Abstract] OR LR-TIV[Title/Abstract] OR LR-M[Title/Abstract] OR LR1[Title/Abstract] OR LR2[Title/Abstract] OR LR3[Title/Abstract] OR LR4[Title/Abstract] OR LR5[Title/Abstract] OR LRTIV[Title/Abstract] OR LRM[Title/Abstract]))) OR Radiology Information Systems[Title/Abstract])) AND ((hepatocellular carcinoma*[Title/Abstract] OR hepatocellular neoplasm*[Title/Abstract] OR hepatocellular cancer[Title/Abstract] OR hepatic cell carcinoma*[Title/Abstract] OR HCC[Title/Abstract] OR cholangiocarcinoma*[Title/Abstract] OR hepatic nodule*[Title/Abstract] OR liver lesion*[Title/Abstract] OR liver nodule*[Title/Abstract] OR liver neoplasm*[Title/Abstract]))) |
| Embase | 1.(‘LI-RADS’ or ‘LIRADS’):ab,ti,kw  2.‘Liver Imaging Reporting and Data System’:ab,ti,kw  3. (‘LR-1’ or ‘LR-2’ or ‘LR-3’ or ‘LR-4’ or ‘LR-5’ or ‘LR-5V’ or ‘LR-TIV’ or ‘LR-M’ or LR1 or LR2 or LR3 or LR4 or LR5 or LRTIV or LRM ):ab,ti,kw  4. (‘Carcinoma, Hepatocellular’ or Cholangiocarcinoma):ab,ti,kw  5. (‘hepatocellular carcinoma*’ or ‘hepatocellular neoplasm*’ or ‘hepatocellular cancer’ or ‘hepatic cell carcinoma*’ or HCC or cholangiocarcinoma* or ‘hepatic nodule*’ or ‘liver lesion*’ or ‘liver nodule*’ or ‘liver neoplasm*’ ):ab,ti,kw  6. or /1-3  7. 4 or 5  8.6 and 7 |
| Cochrane Central Register of Controlled Trials | 1. (‘LI-RADS’ or ‘LIRADS’):ab,ti,kw  2. ‘Liver Imaging Reporting and Data System’:ab,ti,kw  3. (‘LR-1’ or ‘LR-2’ or ‘LR-3’ or ‘LR-4’ or ‘LR-5’ or ‘LR-5V’ or ‘LR-TIV’ or ‘LR-M’ or LR1 or LR2 or LR3 or LR4 or LR5 or LRTIV or LRM ):ab,ti,kw  4. (‘Carcinoma, Hepatocellular’ or Cholangiocarcinoma):ab,ti,kw  5. (‘hepatocellular carcinoma*’ or ‘hepatocellular neoplasm*’ or ‘hepatocellular cancer’ or ‘hepatic cell carcinoma*’ or HCC or cholangiocarcinoma* or ‘hepatic nodule*’ or ‘liver lesion*’ or ‘liver nodule*’ or ‘liver neoplasm*’ ):ab,ti,kw  6. or /1-3  7. 4 or 5  8. 6 and 7 |

Table 2: Characteristics of the included articles

|  | Background | | | Patients | | | | Index test | | Reference standard | | | Observations | | | |
| --- | --- | --- | --- | --- | --- | --- | --- | --- | --- | --- | --- | --- | --- | --- | --- | --- |
| Study | Year | Country | Study design | N | Age ,y  Mean/median | SD/range | Men, % | Index test | LI-RADS version | HCC | Non-HCC  malignancy | Benign | n | HCC | Non-HCC  malignancy | Benign |
| Schellhaas B.[13] | 2017 | Germany | Prospective | 100 | 66.1 | 42-85 | 85 | CEUS | 2016 | Pathology or CCRS | Pathology | Pathology or CCRS | 100 | 87 | 6 | 7 |
| Terzi E.[15] | 2017 | Italy | Retrospective | 848 | 70 | 31-89 | 54 | CEUS | 2016 | Pathology or CCRS | Pathology | Pathology or CCRS | 1006 | 820 | 53 | 133 |
| Tan Z.[16] | 2020 | Singapore | Retrospective | 45 | 63.1 | 34-84 | 71.1 | CEUS | 2017 | Pathology or CCRS | NA | Pathology or CCRS | 46 | 37 | 0 | 9 |
| Zheng W.[17] | 2019 | China | Retrospective | 1826 | 54 | 44-62 | 90 | CEUS | 2017 | Pathology or CCRS | Pathology | Pathology or CCRS | 2020 | 1514 | 138 | 368 |
| Wang JY.[18] | 2020 | China | Retrospective | 258 | 52 | 11 | 78 | CEUS | 2017 | Pathology or CCRS | Pathology | Pathology or CCRS | 355 | 115 | 5 | 235 |
| Li J.[19] | 2019 | China | Retrospective | 1366 | 52.3 | 12.0 | 80.3 | CEUS | 2017 | Pathology | Pathology | Pathology | 1366 | 985 | 139 | 242 |
| Ding J.[20] | 2020 | China | Retrospective | 239 | 59.1 | 8.0 | 76.2 | CEUS And CT/MRI | 2017 | Pathology | Pathology | Pathology | 273 | 225 | 22 | 26 |
| Makoyeva.A[47] | 2020 | Canada | Retrospective | 184 | 62 | 27-87 | 75 | CEUS | 2017 | Pathology or CCRS | Pathology | Pathology or CCRS | 196 | 139 | 18 | 39 |
| Ghiuchici [14] | 2021 | Romnia | Retrospective | 382 | 63 | 9.6 | 68.3 | CEUS | 2017 | Pathology or CCRS | Pathology | Pathology or CCRS | 464 | 359 | 68 | 37 |
| Ciocalteu A[48] | 2021 | Romnia | Retrospective | 143 | 65.7 | 8.2 | 67.8 | CEUS | 2017 | Pathology or CCRS | Pathology | Pathology or CCRS | 191 | 141 | 18 | 32 |
| Xian MF[49] | 2021 | China | Retrospective | 174 | 53 | NA | 83.3 | CEUS | 2017 | Pathology or CCRS | Pathology | Pathology or CCRS | 174 | 142 | 25 | 7 |
| Ding J.(2)[50] | 2021 | China | Retrospective | 264 | 59.4 | 9.3 | 76.5 | CEUS | 2017 | Pathology | Pathology | Pathology | 264 | 223 | 23 | 18 |
| Yang D.[51] | 2021 | China | Retrospective | 205 | 52 | 19-83 | 79.0 | CEUS | 2017 | Pathology or CCRS | Pathology | Pathology or CCRS | 205 | 142 | 26 | 37 |
| Zeng D[52] | 2021 | China | Retrospective | 289 | 47 | 20-79 | 94.5 | CEUS | 2017 | Pathology or CCRS | Pathology | Pathology or CCRS | 315 | 286 | 22 | 7 |
| Lv K[53] | 2021 | China | Retrospective | 250 | 61.3 | 10.8 | 74.4 | CEUS And CT/MRI | 2017/2018 | Pathology | Pathology | Pathology | 259 | 172 | 61 | 26 |
| Basha(1)[21] | 2017 | Egypt | Retrospective | 55 | 58.8 | 10.8 | 58.2 | CT | 2014 | Pathology | Pathology | Pathology | 55 | 34 | 2 | 19 |
| Cha[22] | 2017 | Koera | Retrospective | 421 | 57 | 20-82 | 72 | CT/MRI | 2014 | Pathology | Pathology | Pathology | 445 | 397 | 31 | 17 |
| Choi[23] | 2016 | Koera | Retrospective | 294 | 56.5 | 9.4 | 83.7 | MRI | 2014 | Pathology or CCRS | Pathology | Pathology or CCRS | 379 | 327 | 9 | 43 |
| Fraum[24] | 2018 | USA | Retrospective | 178 | 60.3 | 9.7 | 74.2 | CT/MRI | 2014 | Pathology | Pathology | Pathology | 220 | 136 | 42 | 42 |
| Kim BR.[25] | 2017 | Koera | Retrospective | 68 | 57.2 | 9.0 | 69.1 | CT/MRI | 2014 | Pathology | NA | Pathology | 112 | 75 | 0 | 37 |
| Kim YY.[26] | 2018 | Koera | Retrospective | 143 | 58.0 | 32-81 | 83.4 | MRI | 2014 | Pathology or CCRS | Pathology | Pathology or CCRS | 202 | 129 | 6 | 67 |
| Liu W.[27] | 2018 | China | Retrospective | 249 | 51 | 26-79 | 85.5 | CT/MRI | 2014 | Pathology | Pathology | Pathology or CCRS | 297 | 178 | 13 | 106 |
| An C.[28] | 2020 | Koera | Retrospective | 217 | 59 | 36-85 | 76.5 | CT/MRI | 2014 | Pathology | Pathology | Pathology | 231 | 114 | 58 | 59 |
| Qi Z.[29] | 2016 | China | Retrospective | 178 | NR | NR | NR | MRI | 2014 | Pathology | Pathology | Pathology | 192 | 138 | 0 | 54 |
| Joo I.[30] | 2018 | Koera | Retrospective | 288 | NR | NR | NR | MRI | 2017 | Pathology or CCRS | Pathology | Pathology or CCRS | 387 | 292 | 15 | 80 |
| Rosiak[31] | 2018 | Poland | Retrospective | 32 | NR | NR | 75 | MRI | 2017 | Pathology | NA | Pathology | 70 | 50 | 0 | 20 |
| Renzulli[32] | 2018 | Italy | Prospective | 228 | 63.7 | 10.6 | 79.4 | MRI | 2017 | Pathology or CCRS | Pathology | Pathology or CCRS | 420 | 347 | 4 | 69 |
| Min JH.[33] | 2018 | Koera | Prospective | 91 | 59 | 32-76 | 83.5 | MRI | 2017 | Pathology | Pathology | Pathology or CCRS | 117 | 95 | 3 | 19 |
| Alhasan[34] | 2019 | Canada | Retrospective | 59 | 63.2 | 10.7 | 76.3 | CT | 2017 | Pathology or CCRS | Pathology | Pathology or CCRS | 104 | 75 | 4 | 25 |
| Zhang T.[35] | 2019 | China | Retrospective | 203 | 50.3 | 10.9 | 77.3 | MRI | 2017 | Pathology | Pathology | Pathology or CCRS | 245 | 165 | 30 | 50 |
| Basha(2)[36] | 2019 | Egypt | Prospective | 165 | 55.6 | 9.6 | 77 | MRI | 2017 | Pathology | NA | Pathology | 188 | 67 | 0 | 121 |
| Ren AH.[37] | 2019 | Koera | Retrospective | 181 | 56 | 30-77 | 78 | MRI | 2018 | Pathology or CCRS | Pathology | Pathology or CCRS | 217 | 146 | 16 | 55 |
| Lee SY.[38] | 2019 | Koera | Retrospective | 298 | 57.4 | 9.7 | 72 | MRI | 2018 | Pathology | Pathology | Pathology or CCRS | 382 | 286 | 33 | 63 |
| Chernyak[39] | 2019 | USA | Retrospective | NR | NR | NR | NR | CT/MRI | 2018 | Pathology | Pathology | Pathology | 68 | 56 | 0 | 12 |
| Gaetano[40] | 2019 | Italy | Retrospective | 37 | 64.0 | 10.2 | 75 | MRI | 2018 | Pathology | NA | Pathology | 43 | 17 | 0 | 26 |
| Jiang H.[41] | 2019 | China | Prospective | 211 | NR | NR | 80 | MRI | 2018 | Pathology | Pathology | Pathology | 229 | 173 | 32 | 24 |
| Lee SM.[42] | 2019 | Koera | Retrospective | 387 | 59.0 | 10.0 | 79 | MRI | 2018 | Pathology | Pathology | Pathology or CCRS | 422 | 234 | 45 | 143 |
| Barabino[43] | 2020 | Italy | Retrospective | 40 | 66.6 | NR | 77.5 | CT/MRI | 2018 | Pathology | Pathology | Pathology | 40 | 33 | 5 | 2 |
| Cannella[44] | 2019 | Italy | Retrospective | 155 | 57.2 | 10.3 | 69.7 | MRI | 2018 | Pathology or CCRS | Pathology | Pathology or CCRS | 205 | 126 | 0 | 79 |
| Kierans[45] | 2020 | USA | Retrospective | 122 | 56.5 | 21-97 | 66.4 | MRI | 2018 | Pathology or CCRS | Pathology | Pathology or CCRS | 159 | 84 | 13 | 62 |
| A.Ko[46] | 2020 | korea | Retrospective | 117 | 57.9 | 9.5 | 88.9 | MRI | 2018 | Pathology | Pathology | Pathology or CCRS | 137 | 89 | 16 | 32 |
| Zhang Y. | 2021 | China | Retrospective | 235 | 51.4 | 11.3 | 85.5 | MRI | 2018 | Pathology or CCRS | Pathology | Pathology or CCRS | 250 | 196 | 15 | 39 |
| Xie S. | 2021 | China | Retrospective | 133 | 52.0 | 44-59 | 97.0 | MRI | 2018 | Pathology or CCRS | Pathology | Pathology or CCRS | 174 | 98 | 6 | 70 |

Table 3: Detailed information of the excluded studies

| **Author** | **Title** | **Journal** | **Year** | **Volume and page** |
| --- | --- | --- | --- | --- |
| **Reason for excluding: only including patients with HCC and non-HCC malignancies** | | | | |
| Huang JY. et al. | Can contrast enhanced ultrasound differentiate intrahepatic cholangiocarcinoma from hepatocellular carcinoma? | World journal of gastroenterology | 2020 | 27: 3938-3951 |
| Chen LD. et al. | Comparison between M-score and LR-M in the reporting system of contrast-enhanced ultrasound LI-RADS | European radiology | 2019 | 8: 4249-4257 |
| Jeon, S. K.  et al. | Combined hepatocellular cholangiocarcinoma: LI-RADS v2017 categorisation for differential diagnosis and prognostication on gadoxetic acid-enhanced MR imaging | European radiology | 2019 | 1:373-382 |
| Choi, S. H.  et al. | LI-RADS Classification and Prognosis of Primary Liver Cancers at Gadoxetic Acid–enhanced MRI | Radiology | 2019 | 3:388-397 |
| Ludwig, D. R. et al. | Hepatocellular carcinoma (HCC) versus non-HCC: accuracy and reliability of Liver Imaging Reporting and Data System v2018 | Abdominal radiology (New York) | 2019 | 6:2116-2132 |
| Seo,N.  et al. | Hepatic sarcomatoid carcinoma: magnetic resonance imaging evaluation by using the liver imaging reporting and data system | European radiology | 2019 | 7:3761-3771 |
| Kim,Y.Y.  et al. | Hepatocellular Carcinoma versus Other Hepatic Malignancy in Cirrhosis: Performance of LI-RADS Version 2018 | Radiology | 2019 | 1:72-80 |
| Horvat, N. et al. | Imaging features of hepatocellular carcinoma compared to intrahepatic cholangiocarcinoma and combined tumor on MRI using liver imaging and data system (LI-RADS) version 2014 | Abdominal radiology (New York) | 2018 | 1:169-178 |
| Joo, I.  et al. | Diagnostic accuracy of liver imaging reporting and data system (LI-RADS) v2014 for intrahepatic mass-forming cholangiocarcinomas in patients with chronic liver disease on gadoxetic acid-enhanced MRI | Journal of magnetic resonance imaging | 2016 | 5:1330-1338 |
| Zheng W.  et al. | Added-value of ancillary imaging features for differentiating hepatocellular carcinoma from intrahepatic mass-forming cholangiocarcinoma on Gd-BOPTA-enhanced MRI in LI-RADS M | Abdominal radiology (New York) | 2021 | Online ahead of print |
| Leonardo C. et al. | From LI-RADS Classification to HCC Pathology: A Retrospective Single-Institution Analysis of Clinico-Pathological Features Affecting Oncological Outcomes after Curative Surgery | Diagnostics (Basel) | 2022 | Online ahead of print. |
| Guo HL.  et al. | Contrast-Enhanced Ultrasound for Differentiation Between Poorly Differentiated Hepatocellular Carcinoma and Intrahepatic Cholangiocarcinoma | Journal of ultrasound in the medicine | 2021 | Online ahead of print |
| Choi SH.  et al. | Radio-pathologic correlation of biphenotypic primary liver cancer (combined hepatocellular cholangiocarcinoma): changes in the 2019 WHO classification and impact on LI-RADS classification at liver MRI | European radiology | 2021 | 31(12):9479-9488 |
| Liang YY.  et al. | Liver Imaging and Data System (LI-RADS) Version 2018 and Other Imaging Features in Intrahepatic Cholangiocarcinoma in Chinese Adults with vs. without Chronic Hepatitis B Viral Infection | Canadian Journal of Gastroenterology & Hepatology | 2021 | 2021:6639600 |
| Huang Z.  et al. | MR versus CEUS LI-RADS for Distinguishing Hepatocellular Carcinoma from other Hepatic Malignancies in High-Risk Patients | Ultrasound in Medicine & Biology | 2021 | 47(5):1244-1252 |
| **Reason for excluding: insufficient data for the analysis of proportions of HCC, overall malignancy in each LI-RADS category and sensitivity and specificity of LR-5/M** | | | | |
| Kang HJ et al. | Additional value of contrast-enhanced ultrasound (CEUS) on arterial phase non-hyperenhancement observations (>= 2 cm) of CT/MRI for high-risk patients: focusing on the CT/MRI LI-RADS categories LR-3 and LR-4 | Abdominal Radiology | 2020 | 45:55-63 |
| Schellhaas B. et al. | Contrast-Enhanced Ultrasound Algorithms (CEUS-LIRADS/ESCULAP) for the Noninvasive Diagnosis of Hepatocellular Carcinoma - A Prospective Multicenter DEGUM Study | Ultraschall in der Medizin | 2020 | Online ahead of print. |
| Zhou H. et al. | Contrast-Enhanced Ultrasound Liver Imaging Reporting and Data System in Diagnosing Hepatocellular Carcinoma: Diagnostic Performance and Interobserver Agreement | Ultraschall in der Medizin | 2020 | Online ahead of print. |
| Albrecht, H. et al. | Diagnostic accuracy of the liver imaging reporting and data system (LI-RADS) for hepatic nodules in cirrhotic patients: A 2 year retrospective analysis | Laboratory Investigation | 2016 | 413A-414A |
| Bae, J. S. | Diagnostic accuracy of gadoxetic acid-enhanced MR for small hypervascular hepatocellular carcinoma and the concordance rate of Liver Imaging Reporting and Data System (LI-RADS) | PLoS ONE | 2017 | 5:e0178495 |
| Choi, S. H.  et al. | Liver Imaging Reporting and Data System v2014 With Gadoxetate Disodium-Enhanced Magnetic Resonance Imaging: Validation of LI-RADS Category 4 and 5 Criteria | Investigative radiology | 2016 | 8:483-490 |
| Fowler, K. J. et al. | Interreader reliability of LI-RADS version 2014 algorithm and imaging features for diagnosis of hepatocellular carcinoma: A large international multireader study | Radiology | 2018 | 1:173-185 |
| Tanabe, M.  et al. | Imaging Outcomes of Liver Imaging Reporting and Data System Version 2014 Category 2, 3, and 4 Observations Detected at CT and MR Imaging | Radiology | 2016 | 1:129-139 |
| Kim, D. H.  et al. | Arterial subtraction images of gadoxetate-enhanced MRI improve diagnosis of early-stage hepatocellular carcinoma | Journal of hepatology | 2019 | 3:534-542 |
| Kim YC et al | Intra-individual comparison of gadolinium-enhanced MRI using pseudo-golden-angle radial acquisition with gadoxetic acid-enhanced MRI for diagnosis of HCCs using LI-RADS | European Radiology | 2019 | 29:2058-2068 |
| Lee HS et al | How to utilize LR-M features of the LI-RADS to improve the diagnosis of combined hepatocellular-cholangiocarcinoma on gadoxetate-enhanced MRI? | European Radiology | 2019 | 29:2408-2416 |
| Min JH.  et al. | A modified LI-RADS: targetoid tumors with enhancing capsule can be diagnosed as HCC instead of LR-M lesions | European Radiology | 2022 | Online ahead of print. |
| Min JH.  et al. | EASL versus LI-RADS: Intra-individual comparison of MRI with extracellular contrast and gadoxetic acid for diagnosis of small HCC | Liver International | 2021 | 41(12):2986-2996 |
| Jiang H.  et al. | Data-Driven Modification of the LI-RADS Major Feature System on Gadoxetate Disodium-Enhanced MRI: Toward Better Sensitivity and Simplicity | Journal of magnetic resonance imaging | 2021 | Online ahead of print. |
| Jeon SK.  et al. | LI-RADS v2018: how to appropriately use ancillary features in category adjustment from intermediate probability of malignancy (LR-3) to probably HCC (LR-4) on gadoxetic acid-enhanced MRI | European Radiology | 2021 | Online ahead of print. |
| Strobel D.  et al. | Real-life assessment of standardized contrast-enhanced ultrasound (CEUS) and CEUS algorithms (CEUS LI-RADS®/ESCULAP) in hepatic nodules in cirrhotic patients—a prospective multicenter study | European Radiology | 2021 | 31(10): 7614–7625 |
| Pan JM.  et al. | Tumor size-based validation of contrast-enhanced ultrasound liver imaging reporting and data system (CEUS LI-RADS) 2017 for hepatocellular carcinoma characterizing | The British journal of radiology | 2021 | 1;94(1126):20201359 |
| **Reason for excluding: including duplicate data** | | | | |
| Huang JY. et al.. | Diagnostic Accuracy of CEUS LI-RADS for the Characterization of Liver Nodules 20 mm or Smaller in Patients at Risk for Hepatocellular Carcinoma | Radiology | 2020 | 2:329-339 |
| Ling W. et al. | The preliminary application of liver imaging reporting and data system (LI-RADS) with contrast-enhanced ultrasound (CEUS) on small hepatic nodules (≤ 2cm) | Journal of Cancer | 2018 | 16:2946-2952 |
| Schellhaas B. et al. | Interobserver Agreement for Contrast-Enhanced Ultrasound (CEUS)-Based Standardized Algorithms for the Diagnosis of Hepatocellular Carcinoma in High-Risk Patients | Ultraschall in der Medizin | 2018 | 6:667-674 |
| Schellhaas B. et al. | Interobserver and intermodality agreement of standardized algorithms for non-invasive diagnosis of hepatocellular carcinoma in high-risk patients: CEUS-LI-RADS versus MRI-LI-RADS | Abdominal Radiology | 2018 | 28:4254-4264 |
| Terzi E.. et al. | CEUS LI-RADS are effective in predicting the risk hepatocellular carcinoma of liver nodules | Digestive and Liver Disease | 2017 | 1:e22 |
| Wang JY. Et al. | Comparison of Contrast-Enhanced Ultrasound versus Contrast-Enhanced Magnetic Resonance Imaging for the Diagnosis of Focal Liver Lesions Using the Liver Imaging Reporting and Data System | Ultrasound in Medicine and Biology | 2020 | 5:1216-1223 |
| Zheng W. et al. | Contrast enhanced ultrasound liver imaging reporting and data system version 2016: Application of the diagnostic algorithm and feedback on 1,773 liver lesions | Ultrasound in Medicine and Biology | 2017 | 43:S41-S42 |
| An.C.  et al. | Curative Resection of Single Primary Hepatic Malignancy: Liver Imaging Reporting and Data System Category LR-M Portends a Worse Prognosis | American journal of roentgenology | 2017 | 3:576-583 |
| Kierans, A. S. et al. | Validation of Liver Imaging Reporting and Data System 2017 (LI-RADS) Criteria for Imaging Diagnosis of Hepatocellular Carcinoma | Journal of magnetic resonance imaging | 2019 | 7:e205-e215 |
| Staphan A.  et al. | Diagnostic accuracy of contrast-enhanced ultrasound algorithm (ACR CEUS LI-RADSV 2016) for the diagnosis of hepatocellular carcinoma in patients with chronic liver disease. | Journal of Gastrointestinal and Liver Diseases | 2018 | 27 (39-40) Supplement 1 |
| Ren AH.  et al. | Ren AH, Xu H, Yang DW, et al. Systematic Training of Liver Imaging Reporting and Data System Magnetic Resonance Imaging v2018 can Improve the Diagnosis of Hepatocellular Carcinoma for Different Radiologists. J Clin Transl Hepatol. 2021;9(4):537-544. doi:10.14218/JCTH.2021.00180 | Journal of Clinical and Translational Hepatology | 2021 | 9(4):537-544 |
| Lee SM.  et al. | Diagnostic Performance of 2018 KLCA-NCC Practice Guideline for Hepatocellular Carcinoma on Gadoxetic Acid-Enhanced MRI in Patients with Chronic Hepatitis B or Cirrhosis: Comparison with LI-RADS Version 2018 | Korean Journal of radiology | 2021 | 22(7):1066-1076. |

Table 4: Distribution of HCC in CEUS and CT/MRI LR-M categories

| HCC, n (%) | LR-1 | LR-2 | LR-3 | LR-4 | LR-5 | LR-M | LR-TIV |
| --- | --- | --- | --- | --- | --- | --- | --- |
| CEUS | 0 (0) | 2 (0.1) | 172 (3.2) | 580(10.8) | 3701(68.5) | 852(15.8) | 93(1.7) |
| CT/MRI | 0 (0) | 15 (0.3) | 331 (7.3) | 926(20.4) | 3030(66.7) | 168(3.7) | 73(1.6) |
| P | 1 | ＜0.01 | ＜0.01 | ＜0.01 | 0.27 | ＜0.01 | 0.66 |

Table 5: Distribution of non-HCC malignancies in CEUS and CT/MRI LR-M categories

| OM n (%) | LR-1 | LR-2 | LR-3 | LR-4 | LR-5 | LR-M | LR-TIV |
| --- | --- | --- | --- | --- | --- | --- | --- |
| CEUS | 0 (0) | 0(0) | 13(2.4) | 14(2.6) | 82(15.1) | 427(78.6) | 7(1.3) |
| CT/MRI | 0 (0) | 2(0.5) | 13(3.1) | 42(9.9) | 78(18.3) | 263(61.7) | 28(6.6) |
| P | 1 | 0.11 | 0.53 | ＜0.01 | 0.22 | ＜0.01 | ＜0.01 |
